# X chromosome inactivation in a female carrier of a 1.28 Mb deletion encompassing the human X inactivation centre

**DOI:** 10.1098/rstb.2016.0359

**Published:** 2017-09-25

**Authors:** B. de Hoon, Erik Splinter, B. Eussen, J. C. W. Douben, E. Rentmeester, M. van de Heijning, J. S. E. Laven, J. E. M. M. de Klein, J. Liebelt, J. Gribnau

**Affiliations:** 1 Department of Developmental Biology, Rotterdam, The Netherlands; 2 Department of Clinical Genetics, Rotterdam, The Netherlands; 3 Department of Obstetrics and Gynaecology, Erasmus MC, Rotterdam, The Netherlands; 4 Cergentis B.V., Utrecht, The Netherlands; 5 Division of Genetics and Molecular Pathology, Women's and Children's Hospital, North Adelaide, South Australia, Australia

**Keywords:** X chromosome inactivation, deletion, XIC, *XIST*

## Abstract

X chromosome inactivation (XCI) is a mechanism specifically initiated in female cells to silence one X chromosome, thereby equalizing the dose of X-linked gene products between male and female cells. XCI is regulated by a locus on the X chromosome termed the X-inactivation centre (XIC). Located within the XIC is *XIST*, which acts as a master regulator of XCI. During XCI, *XIST* is upregulated on the inactive X chromosome and chromosome-wide *cis* spreading of *XIST* leads to inactivation. In mouse, the Xic comprises *Xist* and all *cis*-regulatory elements and genes involved in *Xist* regulation. The activity of the XIC is regulated by *trans*-acting factors located elsewhere in the genome: X-encoded XCI activators positively regulating XCI, and autosomally encoded XCI inhibitors providing the threshold for XCI initiation. Whether human XCI is regulated through a similar mechanism, involving *trans*-regulatory factors acting on the XIC has remained elusive so far. Here, we describe a female individual with ovarian dysgenesis and a small X chromosomal deletion of the XIC. SNP-array and targeted locus amplification (TLA) analysis defined the deletion to a 1.28 megabase region, including *XIST* and all elements and genes that perform *cis*-regulatory functions in mouse XCI. Cells carrying this deletion still initiate XCI on the unaffected X chromosome, indicating that XCI can be initiated in the presence of only one XIC. Our results indicate that the *trans*-acting factors required for XCI initiation are located outside the deletion, providing evidence that the regulatory mechanisms of XCI are conserved between mouse and human.

This article is part of the themed issue ‘X-chromosome inactivation: a tribute to Mary Lyon’.

## 1. Introduction

X chromosome inactivation (XCI) is a process that takes place in all female somatic cells, and results in almost complete transcriptional silencing of one of the X chromosomes. This process ensures that female XX cells have an equal dosage of X-chromosomal gene products compared to male XY cells [1]. XCI is initiated early during embryogenesis, where all cells of the female embryo proper randomly initiate inactivation of either the paternal or the maternal X chromosome. After the inactive state is established, the inactive X (Xi) is clonally passed on to all daughter cells. As a result, all females are a mosaic of cells with an active paternal X chromosome and cells with an active maternal X chromosome. The X inactivation ratio (XIR) between cells carrying an active paternal X chromosome and cells carrying an active maternal X chromosome differs between female individuals. In a female population the average XIR is 50 : 50, but ranges up to 100 : 0, as a result of chance, genetic predisposition or cell selection [2]. Within a female individual the XIR correlates between tissues of different origin [3].

The genetic elements involved in regulating XCI have been a subject of study for several decades. A region on the X chromosome was identified to be required for XCI. In human, this X inactivation centre (XIC) has been mapped to a 680–1200 kb region at chromosome Xq13 [4–6], deduced from several inversions and truncations involving the X chromosome in different individuals.

The genes that constitute the XIC are conserved in eutherians, and have been mostly studied in mouse. In contrast to the human XIC, the mouse Xic is less well defined and has been mapped to a 10–20 Mb region on the X chromosome [7,8]. Centrally located in the XIC/Xic is the long non-coding RNA (lncRNA) *XIST/Xist*, which is essential for XCI [9,10]. Initiation of XCI is characterized by *XIST/Xist* upregulation on the future Xi, resulting in the *cis* spreading of *XIST/Xist* RNA, which recruits chromatin remodelling complexes that silence the X chromosome [11]. In mouse, several other lncRNA genes located within the Xic are involved in regulating XCI, through co-activation and -repression mechanisms acting to regulate *Xist*, predominantly *in-cis* [12–16]. Several *trans*-acting X-encoded XCI activators ensure that XCI is only initiated in the presence of two X chromosomes [17]. In mouse the RING finger protein 12 (*Rnf12*, also known as RING finger LIM domain interacting protein *Rlim*) represents the most prominent XCI activator [18,19]. *Rnf12* encodes an E3-ubiquitin ligase that targets REX1, a repressor of *Xist*, for proteasomal degradation [20]. Only in cells with more than one X chromosome present in the nucleus is the concentration of RNF12 and other XCI activators high enough to initiate XCI.

In human, the regulation of XCI is less clear. Although the XIC has been localized, the requirement for *trans*-regulatory mechanisms has not been studied. To examine whether XCI in human is also regulated by *trans*-regulatory mechanisms acting on the XIC, which contains all the *cis*-regulatory elements to direct *XIST* expression, we have identified and studied a female carrier with a deletion of the XIC.

## 2. Results

**Figure 1.**
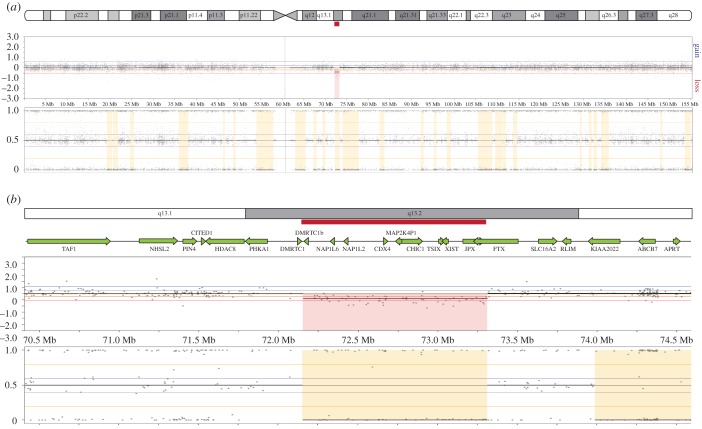
XIC deletion identified by SNP array analysis. (*a*) SNP array analysis showing chromosome X with the XIC deletion. At the top a schematic representation of the X chromosome is shown with the deletion indicated in red. Below the results of the SNP array analysis are shown. Every dot is representing a probe along the X chromosome. The upper panel shows the deleted area in red. The lower panel shows SNP array data revealing loss of *heterozygosity for several regions on the X, of which one overlaps with the deleted sequence* (areas marked in yellow, with B allele frequency (BAF) of 100% or 0%). (*b*) A magnification of the region encompassing the XIC. At the top a schematic representation of the region is shown, with the genes located in this region below. The deletion is indicated in red. Panels are similar to those in (*a*).

A 26 year old, intellectually normal, non-dysmorphic woman presented with primary amenorrhoea, and was diagnosed with hypergonadotropic ovarian failure due to ovarian dysgenesis, also known as premature ovarian insufficiency (POI). Blood examination showed increased FSH levels (more than 40 IU l^−1^), with decreased oestrogen (less than 60 pmol l^−1^). Height was between the 75th–90th centile and no stigmata of Turner syndrome were evident on examination. External genitalia were unambiguously female. Breast development, pubic and axillary hair distribution were normal female at the time of examination following several years of oral contraceptive hormone treatment. On transvaginal ultrasound examination a small uterus was visible, measuring 1.9 × 0.9 × 1.4 cm^3^, with hypoplastic endometrium of 0.1 mm. No clear ovarian tissue was identified; however, an area of thickening without antral follicles 0.6 cm in length was seen in the left ovarian fossa and a similar region 1 cm in length, in the right ovarian fossa. karyotype analysis of blood cells showed a normal 46XX karyotype. DNA was isolated from peripheral blood and was used for molecular SNP karyotyping, using the Human OmniExpress-24 SNP-array from Illumina. Several regions showing loss of heterozygosity were identified, which might be explained by a history of parental consanguinity. However, only one single significant copy number change was identified corresponding with a region showing loss of heterozygosity, which was located on chromosome X and included a deletion of the XIC (figure 1). No other copy number changes were identified (electronic supplementary material, figure S1).

**Figure 2.**
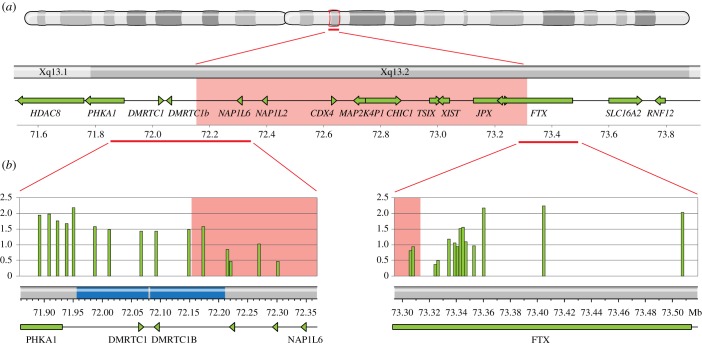
XIC deletion identified PCR. (*a*) The XIC deletion as identified by comparative genomic hybridization (CGH). A schematic representation of the X-chromosome is shown, with the deleted region marked in red. (*b*) Comparison of qPCR results to CGH. A magnification is shown of the regions containing the breakpoints. The top panel shows qPCR results along the breakpoint regions, with copy number plotted against chromosomal position. In the map of the chromosomal region below, the inverted duplication in the centromeric breakpoint region is marked in blue.

**Figure 3.**
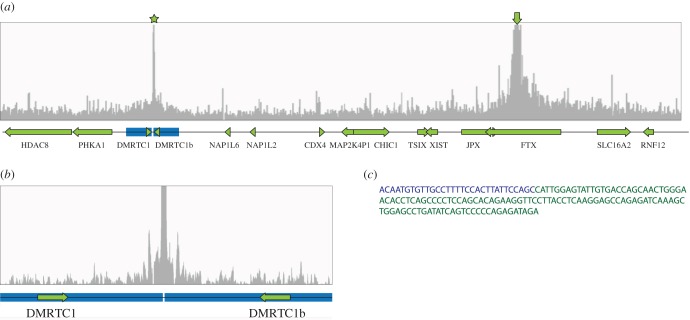
XIC deletion identified by TLA. (*a*) TLA coverage is plotted against chromosomal position. Arrow indicates the location of the primer sets. Star indicates the increase in coverage on the other side of the deleted region. A genetic map of the region is shown below, with the inverted duplication in blue. (*b*) Magnification of the centromeric breakpoint region. (*c*) The break-spanning read, with the sequence centromeric of the breakpoint marked in blue and the sequence telomeric of the breakpoint marked in green.

The centromeric breakpoint of the identified deletion was located in a 148 kb long segment between SNPs rs6650032 and rs6650032. This region is characterized by a large inverted segmental duplication, including *DMRTC1* and *DMRTC1b*, complicating the precise mapping of this breakpoint (figure 2*a*). The location of this breakpoint determines if the duplication is intact and present as two copies of *DMRTC1* and *DMRTC1b*, or partially deleted and consequently present as a single copy of *DMRTC1* on the affected X chromosome. The telomeric breakpoint of this region was located in a 17 kb region, delineated by SNPs rs12688161 and rs5981589. A deletion encompassing the XIC and as small as this, has not been reported before, which prompted us to study this deletion in detail. To characterize the breakpoint regions more precisely we performed qPCR to determine the copy number at consecutive positions along the breakpoint regions. As a control, DNA from a healthy female and a healthy male was used. qPCR analysis along the centromeric breakpoint region, detected two copies centromeric of the inverted duplication in this region, and one copy telomeric. Three qPCR amplicons within the duplication all indicated the presence of 1.5 copies, suggesting the breakpoint is located in the middle of the inverted duplication (figure 2*b*). To map the deletion at the single nucleotide level, targeted locus amplification (TLA) was performed on frozen lymphoblastoid cells. TLA technology entails sequencing of all sequences that occur in physical proximity to a primer pair used for targeted amplification. Using this technology, with primer sets located on the telomeric side of the deletion, we found an increase in coverage in the centre of the inverted duplication, indicating the position of the centromeric breakpoint (figure 3*a*,*b*). Among the obtained reads the break-spanning read was also identified (figure 3*c*). The flanking sequence of the centromeric breakpoint maps to both duplicons of the inverted duplication, but the orientation of the sequence corresponds to the centromeric duplicon. This maps the deletion to ChrX: 72.080.568–73.367.054 (hg19), a region of 1.28 Mb, and among the genes involved in the deletion are *XIST*, *TSIX* and other *cis*-regulatory genes and elements in XCI (electronic supplementary material, table S1).

**Figure 4.**
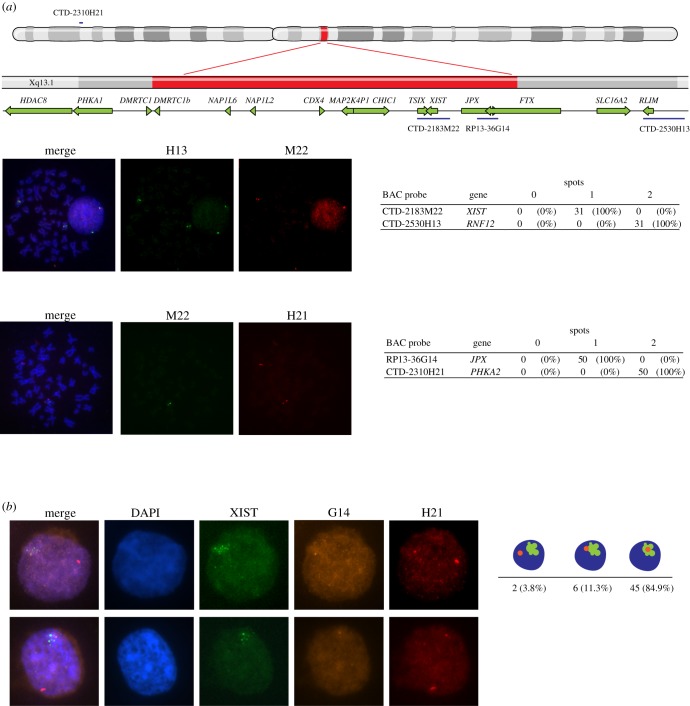
The X-chromosome carrying the deletion is preferentially active. (*a*) At the top, a map of the X-chromosome is shown with the location of the BAC probes that were used indicated in blue. The location of the deletion as identified by CGH and TLA is marked in red. Below, representative images of DNA-FISH for regions located inside or outside the deleted region are shown. BAC CTD-2310H21, containing PHKA2, and BAC CTD 2530H13, containing RNF12, are located outside the deleted region. BAC CTD-2183M22, containing XIST, and BAC RP13-36G14, containing JPX, are located within the deleted region. Results from analysing at least 30 metaphases are shown in the tables to the right. (*b*) Representative pictures of sequential RNA-DNA-FISH. Two representative nuclei are shown with RNA-FISH results for *XIST* RNA in green, and DNA-FISH results for BAC RP13-36G14 in yellow and BAC CTD-2310H21 in red. The table to the right shows the percentage of nuclei with overlapping signals of *XIST* RNA and BAC RP13-36G14 DNA.

The deletion was confirmed by DNA-FISH, using several BACs mapping to different regions of the X chromosome. DNA-FISH using BACs containing *PHKA2* or *RNF12* confirmed the presence of two X chromosomes in all nuclei, while DNA-FISH using BACS covering *XIST* or *JPX* indicated these regions to be present on only one X chromosome in all cells (figure 4*a*). XCI was analysed by *XIST* RNA-FISH on cells from a lymphoblastoid cell line of the patient. All examined cells contained an *XIST* RNA cloud, indicating XCI is initiated in cells with the 1.28 Mb deletion (figure 4*b*). To assess the XIR, allele specific methylation analysis of fragile X mental retardation 1 (FMR1), on DNA isolated from peripheral blood, showed predominant inactivation of one allele (93 : 7%), indicating that XCI is severely skewed in the patient (data not shown). To determine which X chromosome was inactivated, sequential DNA-RNA FISH was performed for *XIST* RNA and the *JPX* locus, included in the deletion. *XIST* RNA domains co-localized with the *JPX* locus of the unaffected X chromosome in most cells, confirming near complete skewing towards inactivation of the unaffected X chromosome (figure 4*b*).

## 3. Discussion

Here we identified a patient with a deletion encompassing the human XIC. Our results demonstrate that in the presence of the identified 1.28 Mb deletion, XCI can still occur on the unaffected X chromosome. Therefore, the region involved in the deletion, extending from *FTX* to the first duplicon of *DMRTC1*, does not affect *trans*-regulation of XCI, but most likely covers most elements and genes involved in *cis*-regulation of human XCI. The identified deletion, and the absence of an X-inactivation phenotype resemble the ΔXTX and Δ(Xite-Dxmit171) deletions that were studied in mouse embryonic stem (ES) cells [16,17]. The ΔXTX deletion involves only *Xist*, *Tsix* and *Xite*, whereas the Δ(Xite-Dxmit171) deletion also includes the neighbouring regulators *Jpx* and *Ftx*. ES cells with heterozygous ΔXTX and Δ(Xite-Dxmit171) deletions, inactivate the wild-type X chromosome, indicating that all *trans*-acting factors are still present. These results and the human deletion presented here indicate that *JPX*, and other sequences including *TSIX*, previously implicated in *trans*-regulation of XCI, through X-pairing and RNA mediated recruitment mechanisms, are not required to properly initiate the human XCI process *in-trans* [13,21].

**Figure 5.**
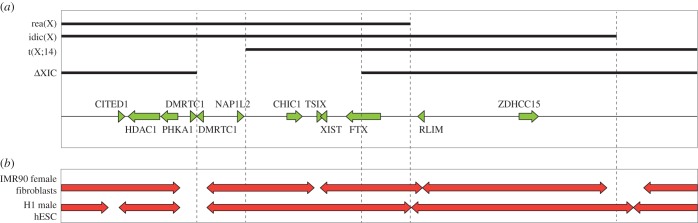
Deletions delineating the human XIC. (*a*) Graph of the chromosomal abnormalities studied to define the XIC. Black bars indicate the sequence present on the abnormal X-chromosome. From top to bottom: the rea(X) of female S.A., the idic(Xp) of female A.G., the X;14 translocation chromosome from a 47XXY male, the XIC deletion reported here. Below a map of the X-chromosomal region Xq13, containing the XIC is shown. (*b*) The topological domain structure of the XIC as identified in female IMR90 fibroblasts and male H1 human ES cells.

Whether the deletion described here represents the entire *cis-*acting XIC remains elusive. This human *cis*-XIC is most likely delineated by the topologically associating domain(s) (TAD) in which XIST resides. TADs were identified by studies examining the higher order chromatin structure, indicating limitation of spatial interactions to *cis* domains of approximately 1 Mb in size [15,22]. Analysis of available data on TADs in human H1 male ES cells and IMR90 female fibroblasts, indicates that the human XIC of H1 ES cells is included in a single large TAD, ranging from *DMRTC1* to *RNF12*, not including *RNF12* [22]. In human IMR90 fibroblasts containing an Xi, this region is divided into two smaller TADs with the boundary at the *XIST/TSIX* locus which now includes *RNF12*, similar to mouse (figure 5). This change in higher order topology might be instructive in or the consequence of XCI and considering these data, the human XIC might extend beyond the studied deletion from *DMRTC1* to *FTX*, and might include *RNF12*.

Our results indicate that the region involved in the deletion does not include important *trans-*acting factors. Although the identity of these factors remains elusive so far, our results are compatible with a role for human *RNF12* in *trans*-activation of human XCI, as *RNF12* is not included in the deletion and still present on both X chromosomes of the female individual described here (figure 3*a*). A role for *RNF12* in human XCI is further supported by a recent finding that female carriers of a mutation in *RNF12* show exclusive XCI of the mutated X [23]. Skewed XCI could be the consequence of cell selection processes, but male carriers with the same mutation are born only displaying X-linked intellectual disability, which might implicate a direct involvement of RNF12 in XCI. This finding is also concordant with mouse studies which show that one functional copy of *Rnf12* is required for establishment of the inactive X chromosome [16]. The XIC deletion identified here is comparable to the region described to represent the human XIC, which was defined by genetic studies involving large X chromosomal abnormalities resulting from truncations and translocations. At first the XIC was delineated by an X;14 translocation in an XXY male and the breakpoint of an isodicentric X chromosome (idic(X)) [4,5,24]. Based on these rearrangements the XIC was estimated to measure at least 0.8 Mb in size with a maximum of 2.89 Mb (figure 5). Later the telomeric boundary was redefined by studies involving another rearranged X chromosome (rea(X)), reducing the estimated size of the XIC to 0.68–1.2 Mb [6]. In all these individuals XCI is still initiated, leading to inactivation of the X;14 translocation product, or the mutated idic(X) and rea(X) X chromosomes, demonstrating the presence of sufficient levels of XCI activators for initiation of XCI. Interestingly, the rea(X) does not contain an intact copy of *RNF12*. These results demonstrate that either *RNF12* does not function as a *trans*-acting activator, or that other human *trans*-acting activators exist next to *RNF12* and are located on the proportion of the X chromosome not deleted from rea(X). In support of this latter hypothesis, mouse studies also hinted at the presence of additional XCI activators besides *Rnf12*, as ES cells carrying a heterozygous *Rnf12* deletion still initiate XCI, although at a reduced frequency [18,19,25]. Although our results suggest RNF12 is a *trans*-acting activator of human XCI and are concordant with mouse studies, further investigation is required.

**Figure 6.**
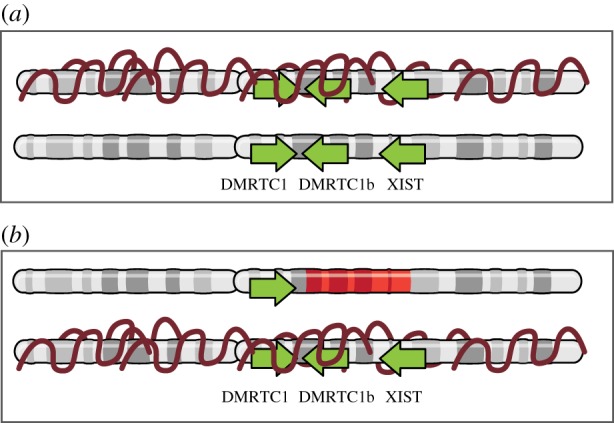
*In-cis* deletion of the *cis*-XIC and *DMRTC1* results in *DMRTC1* dose deficiency. (*a*) Normal situation: a schematic representation of two X chromosomes after X-inactivation is shown. Two copies of *DMRTC1* remain active. (*b*) *In*-*cis* deletion of the XIC and DMRTC1: as a result of the deletion the unaffected X chromosome is preferentially inactivated, and only one copy of *DMRTC1* remains active.

The female individual presented here suffers from ovarian dysgenesis. As our results indicate complete skewing of XCI, with the affected X chromosome remaining active, the deletion itself might be involved in ovarian dysgenesis. Prior work has also implicated chromosome Xq13, harbouring the XIC, in amenorrhoea, as several cases have been described with a breakpoint in this region and primary amenorrhoea and various signs of Turner syndrome [26]. Hypothesizing that one of the genes in the identified deletion is responsible for the ovarian dysgenesis phenotype, we assessed all involved genes by gene ontology (GO) analysis using the AmiGO 2 browser. GO terms involved in gonadal functioning or gonadal development, revealed one candidate gene: DMRT-like family C1 (*DMRTC1*), implicated in sex differentiation, and included in the 1.28 Mb deletion studied here. Little is known about *DMRTC1* itself. It is part of the Doublesex and Mab-3 Related Transcription factor (*DMRT*) gene family, involved in sex-determination in vertebrates. *DMRT1* has been extensively studied, and loss of one copy of human *DMRT1* results in defective testicular development and XY male-to-female sex reversal. No studies regarding *DMRTC1* functioning in human are published. However, of the three mouse homologues of DMRTC1, Dmrt8.3 is expressed in XX germ cells of the embryonic ovary, from embryonic day 13.5 onwards, suggesting involvement in meiosis [27]. Taken together, a role for *DMRTC1* in ovarian development is likely, and it can be hypothesized that the deletion of one of the two copies present on the X chromosome causes ovarian dysgenesis in the individual described here. XCI is 100% skewed and as a result cells carrying the XIC deletion will contain only one active copy of *DMRTC1/DMRTC1b* instead of two, which might result in a dosage deficiency for *DMRTC1/DMRTC1b* (figure 6), leading to ovarian dysgenesis. Interestingly, functional loss of other protein coding genes, including *CDX4* and *CHIC1* appears well tolerated, which might be explained by redundant mechanisms, as reported for *Cdx4* in mice, or escape of XCI.

In summary, our studies show that XCI is normally initiated in a female carrier with a 1.28 Mb deletion of the XIC, and indicate that the *trans*-acting information required for female specific initiation of XCI is located outside the identified deletion. Our findings highlight the evolutionary conservation of pathways and mechanisms involved in regulation of XCI in eutherians.

## 4. Material and methods

### (a) Targeted locus amplification

Cross-linked chromatin, was fragmented and re-ligated, and two sets of primer pairs were designed for the sequence telomeric of the deletion, and used in individual TLA amplifications according to de Vree *et al.* [28]. PCR products were purified and pooled, prepared for sequencing using the Illumina NexteraXT protocol, and sequenced on an Illumina Miseq sequencer. Reads were mapped using a BWA-SW algorithm allowing partial mapping. In case of a deletion, existing interactions with the deleted region are abolished and new interactions with the new neighbouring sequence are produced.

### (b) DNA-FISH

Cells were arrested in metaphase using colcemid for 2 h, treated with 0.075 M KCl, and fixed with methanol/acetic acid. Slides were dehydrated, denatured and incubated with probe mixture as described in [18]. Probes, indicated in figure 4*a*, were detected with a FITC conjugated mouse-anti-digoxigenin antibody or Alexa 594 conjugated streptavidin. Metaphase spreads were first identified in DAPI and subsequently the number of red and green foci were counted.

### (c) DNA-RNA-FISH

Cytospin preparations were prepared and cells were fixed with 4% PFA, and RNA-FISH was performed as described before [17]. Cells were analysed for presence of an *XIST* RNA cloud and photographs were taken. Subsequently slides were subjected to DNA-FISH. Cells were denatured and incubated with probe mixture and detected with a FITC conjugated mouse-anti-digoxigenin antibody or Alexa 594 conjugated streptavidin. Cells analysed for RNA-FISH were re-analysed for a DNA-FISH signal. Pictures from DNA-FISH and RNA-FISH were overlaid and scored for co-localization of signals.

### (d) RT-PCR

Quantitative RT-PCR was performed using Platinum Taq DNA polymerase (Life Technologies), SYBR green (Sigma Aldrich) according to the manufacturer's instructions. Primer sets and their chromosomal locations are listed in electronic supplementary material, Methods 1.

### (e) SNP and CNV detection

Molecular SNP karyotyping was performed on a Human OmniExpress-24 SNP-array (Illumina). Data analysis was performed with the BioDiscovery's SNP-FASST2 segmentation algorithm, an extension of the FASST2 segmentation algorithm (a hidden Markov model [HMM] based approach). B-allele frequency probes were assigned to a range of possible states and a combination of the BAF and log-R states were used to make the final copy number and allelic event calls. The significance threshold for segmentation was set at 5×10^−9^ also requiring a minimum of eight probes per segment and a maximum probe spacing of 1000 kb between adjacent probes before breaking a segment. The log ratio thresholds for single copy gain and single copy loss were set at 0.25 and −0.2, respectively. The homozygous frequency threshold was set to 0.85. The homozygous value threshold was set to 0.8. The heterozygous imbalance threshold was set to 0.4. The minimum loss of heterozygosity length was set at 300 kb.

## Data accessibility

This article has no additional data.
